# Impact of Environmental Factors and Management Practices on Bee Health

**DOI:** 10.3390/insects15120996

**Published:** 2024-12-16

**Authors:** Ivana Tlak Gajger, Franco Mutinelli

**Affiliations:** 1 NRL for Honeybee Diseases, Faculty of Veterinary Medicine, University of Zagreb, Heinzelova 55, 10000 Zagreb, Croatia; 2 NRL for Honey Bee Health, Istituto Zooprofilattico Sperimentale delle Venezie, Viale dell’Università, 10, 35020 Legnaro, Italy; fmutinelli@izsvenezie.it

The honey bee is a symbol of *One Health*, a holistic approach to animal, human and environment health, and beekeeping is an economic branch of exceptional importance for public health [1]. However, in recent years, there has been a lot of discussion about the so-called honey bee health crisis. Consequently, increased attention has been paid to its causes, especially new pathogens, pests, and the geographical redistribution of invasive species, as well as the efficacy of biosecurity measures and practices at the apiary. Here, we outline the different stressors of global importance which are weakening and reducing the number of honeybee and bumblebee colonies [2], as well as *Osmia* spp. solitary bees [3]. Many factors, such as the presence of pathogens, nest destructors, negative environmental drivers, global warming (in particular), agricultural intensification and pesticides, habitat loss, and managing practices, are reported as the main causes of bee depopulation and losses in rearing operations and apiaries. The published articles reflect the complex impacts of environmental factors and management practices on bee health. Herein, we therefore summarize the main focus and results of the twenty-three original articles and one communication that have been published in this Special Issue, the Impact of Environmental Factors and Management Practices on Bee Health. The research has been thematically organized focusing on novel epidemiology studies, diagnostic tools and technologies, monitoring programs, agricultural, beekeeping and veterinary managing practices, biosecurity–control–eradication measures, disinfection methods, and the development of new bee disease control strategies.

The early estimation of infection levels with pathogens and parasites is important to avoid the development of clinical symptoms and further spreading of diseases. The article by Betti et al. [4] described a newly developed multi-scale mathematical model which can investigate the impact of inter-colony interactions on the transmission dynamics of honeybee diseases. The findings indicate that elevated drifting levels significantly enhance the disease spread rate among honey bee colonies. Additionally, the model highlights the necessity for highly efficient worker bees’ guarding behavior to effectively mitigate disease transmission. Notably, in densely populated apiaries, the implications of drifting are of greater concern compared to robbing behavior [4]. In recent decades, the ectoparasitic mite *Varroa destructor* has become a major global threat to managed honey bee (*Apis mellifera*) colonies, and drifting is one of the possibilities for its high reinvasions between colonies and neighboring apiaries. So, an integrated pest management approach without using synthetic acaricides against varroosis motivated Qadir et al. [5] to test the effectiveness of formic acid, oxalic acid, and thymol applied at different concentrations and quantities [5]. Also, essential oils and their components are generally known for their acaricidal effects and are used as an alternative to control the population of *V. destructor* instead of synthetic acaricides. The 30 different essential oils were screened by using a glass-vial residual bioassay, and for essential oils with the best selectivity ratio, their main components were detected and quantified by GC-MS/MS, where the most suitable oils were peppermint and manuka, followed by oregano, litsea, carrot, and cinnamon [6]. Bila Dubaić et al. [7] discussed the possibilities and challenges of detecting and effectively monitoring feral and wild honey bees in urban environments, as well as the role of citizen science in these efforts. Their findings will support ongoing initiatives aimed at better understanding and enhancing naturally selected resistance mechanisms against the *V. destructor* mite. This understanding is expected to help mitigate the current threats to and risks of global beekeeping and food production security. Kovačić et al. [8] investigated the effect of queen caging on honey bee colonies’ post-treatment and the development and optimal timing of methods applied during honey production during the main summer nectar flow in different Mediterranean countries. The study results showed that caging the queen with subsequent oxalic acid treatment 25 days after caging is an efficient method to control *V. destructor*, while the starting point of queen caging concerning the main summer nectar flow affects honey production [8]. Kolics et al. [9] provided basic information about changes in lithium levels in honey bees and their products following anti-varroa treatment and concluded that lithium treatment left combs (beeswax) lithium-free.

Kušar et al. [10] investigated the relationship between clinical symptoms of American foulbrood and spore counts by quantifying spores in honey and hive debris samples from honeybee colonies with known severity of symptoms. For that purpose, they used a newly developed qPCR assay which enables reliable detection and quantification of *P. larvae* in different in-hive sample types. The proper implementation of control measures and effective final disinfection can significantly reduce the recurrence of visible clinical signs of American foulbrood. Tlak Gajger et al. [11] evaluated ten commercially available disinfectants commonly used in beekeeping, along with some that have proven efficacy in the medicinal and veterinary fields, against different strains of the *P. larvae* bacterium.

Glavinić et al. [12] provided basic information on Nosemosis and reviewed currently known control measures (including beekeeping practices and chemical and natural substances), and they analyzed the application of *Agaricus bisporus* water crude extract on honey bees for the first time. The results showed anti-*Nosema ceranae* and nutrigenomic effects of *A. bisporus* extract when supplementation was preventive or in the moment of infection with *N. ceranae* spores. The stress caused by exposure to thiamethoxam and *Nosema* spp. invasion was mitigated by adjustments in the honey bee colony’s dynamics and an increase in the number of worker bees—a behavior known as hormesis. Understanding the factors behind this phenomenon should be included in the prospective risk assessment of plant protection products to enhance the interpretation of field studies and management practices in the future [13].

Mraz et al. [14] studied the prevalence of common honey bee pathogens and viruses in various habitats throughout the Czech Republic. They found that the most prevalent honey bee pathogens belonged to the family Trypanosomatidae, including *Lotmaria passim* and *Crithidia mellificae*, and the most common virus was Deformed Wing Virus, followed by Acute Bee Paralysis Virus. From a location perspective, the highest occurrence of pathogens was observed in urban areas, while fewer pathogens were detected in agroecosystems and the least in national parks. Conversely, the trend for viruses was the opposite.

The Small Hive Beetle (*Aethina tumida* Murray, 1867) is an invasive scavenger of honeybees that is originally endemic in sub-Saharan Africa, and it is regulated internationally to preserve the areas still free from this species. To ensure the reliability of official diagnoses following their introduction, an inter-laboratory comparison was organized to assess the morphological and molecular real-time PCR identification of *A. tumida*. Franco et al. reported the results from 22 official diagnostic laboratories, and the results demonstrated the reliability of the diagnosis, covering the entire analytical process [15].

Pavlović et al. [16] showed differences in the elemental composition of larvae from honey bee colonies with different statuses of chalkbrood disease. Mummies had higher concentrations of macro elements in comparison to typically developed larvae from the same hive, while at the same time, they had much lower concentrations of microelements that have known anti-fungal and antimicrobial activities [16].

Phokasem et al. [17] revealed that the honey bees infected with DWV-A and exposed to thiamethoxam had an increased mortality rate and crippled wings in newly emerged worker bees, and it induced expression of immune gene hymenoptaecin and down-regulation of two apoptosis genes (caspase8-like, caspase9-like genes). 

Mejías et al. [18] examined honey and beeswax samples collected in Chile. They performed chemical profiling to assess phenol content using the Folin–Ciocalteu method, measured antioxidant power through the Ferric Reducing Antioxidant Power Assay (FRAP) and evaluated antiradical activity using the 2,2-Diphenyl-1-picrylhydrazyl Assay (DPPH). Additionally, they tested for pesticide residues using HPLC-MS/MS and GC-MS techniques. The findings mapped the suitability index that ranks areas based on their pesticide-free status and biological quality, and those chemical profiles will assist local beekeepers in obtaining international certifications, particularly for the European Union market [18]. Hýbl et al. [19] reported that polyphenols as food supplements improved the food consumption and longevity of honey bees intoxicated by the pesticide thiacloprid, and the expression level of genes encoding detoxification enzymes was quantified. Raimets et al. [20] reported the possibility of the migration of tebuconazole from wax to royal jelly with a strong dilution effect from the original contamination source. Also, no residues were detected in queen bee larvae nor newly emerged queens, indicating that the migration of tebuconazole did not directly endanger the queen but might affect the homeostasis of developing worker bees. Cabezas et al. [21] evaluated the toxicity of six insecticides on buff-tailed bumblebee workers (*Bombus terrestris*): imidacloprid, thiacloprid, deltamethrin, esfenvalerate, sulfoxaflor and a microbial insecticide based on *Bacillus thuringiensis* toxins, which are present in genetically modified (Bt) maize. The results indicated that some currently used insecticides are more acutely toxic to *B. terrestris* than certain neonicotinoids that have been banned [21].

The results of Vilić et al. [22] showed that RF-EMFs at a frequency of 900 MHz can cause oxidative stress in honey bees, with the larval stage being more sensitive than the pupal stage, and there was no linear relationship between the electric field level and effect in any of the developmental stages.

The first survey on the loss rates of honey bee colonies was reported in 2022/2023 in Ethiopia using COLOSS monitoring survey tools, applying a *face-to-face* interview questionnaire [23].

The spider web and barometer tools enable a comprehensive assessment of the implementation status of biosecurity measures, actions taken to protect the environment where stingless bees thrive, the quality and efficiency of nest management techniques, and the monitoring of the health status in Meliponiculture [24].

Understanding how human activities, such as urbanization and agricultural intensification, affect landscapes and insect pollinators is essential for maintaining a healthy pollination system. In this context, Donkersley et al. [25] reported preliminary results indicating that the foraging and nutrition of *A. cerana japonica* may not be negatively impacted by urban land use. They emphasize the need for future comparative studies between *A. cerana* japonica and *A. mellifera*, as these could reveal resilience in pollinators foraging within their native range [25].

Despite specific limitations, Bontšutšnaja et al. [26] saw strong correlations between microscopy and DNA metabarcoding data used for quantification analyses and the botanical origin of bumble bee-collected pollen for *B. terrestris* species. Also, they concluded that the spring vegetation in southern Estonia can support the development of bumblebee colonies, regardless of the specific landscape structure. Additionally, despite the lack of a dominant natural food source, other bumblebee species—particularly generalists—should also be able to find ample forage during their early development phase.

Elfar et al. [27] examined the proteomic variations and biological activities of bee hemolymphs from four localities in Egypt with differing food diversities. The results showed that worker bees fed only sucrose had the lowest protein concentrations and weak biological activities, while those with access to diverse natural foods had the highest protein levels and significant biological activities [27].

In conclusion, the published articles explore complex factors affecting bee health, emphasizing the global crisis in honeybee and pollinator populations. Key insights include the following:

1. Stressors on Bee Populations: factors such as pathogens, pests (e.g., *V. destructor*), pesticides, habitat loss, and climate change contribute to the decline of bee colonies, including honeybees, bumblebees, and solitary bees.
2. Innovative Control Measures: studies highlight effective methods like queen caging combined with oxalic acid treatments for Varroosis control, essential oils as alternatives to synthetic acaricides, and advancements in diagnostic tools like qPCR assays for diseases such as American foulbrood.
3. Impact of Environmental and Agricultural Practices: urbanization, pesticide exposure, and agricultural intensification affect bees differently. For instance, urban areas showed higher pathogen prevalence, while rural areas had more pesticide-related impacts.
4. Chemical and Biological Research: research identified effective natural remedies like *Agaricus bisporus* extract for bee health and studied pesticide residues’ effects on honey and beeswax.
5. Behavioral and Genetic Studies: studies on disease transmission behaviors, genetic responses to stressors, and the influence of food diversity reveal how bees adapt to environmental challenges and human activities.
6. Global and Local Initiatives: efforts like citizen science for monitoring wild bees, the development of biosecurity measures, and comparative studies on bee species in diverse habitats aim to mitigate risks and improve resilience.

The compilation of twenty-three original articles and a communication underscores the urgency of interdisciplinary approaches to safeguard bee health and ecosystem sustainability.
